# An on-board 2G HTS magnets system with cooling-power-free and persistent-current operation for ultrahigh speed superconducting maglevs

**DOI:** 10.1038/s41598-019-48136-x

**Published:** 2019-08-14

**Authors:** Fangliang Dong, Zhen Huang, Luning Hao, Xiaoyong Xu, Zhijian Jin, Nan Shao

**Affiliations:** 1 School of Electronic Information and Electrical Engineering, Shanghai Jiao Tong University, Shanghai, 200240 China; 2 CRRC Changchun Railway Vehicles Co., Ltd, Changchun, 130062 China

**Keywords:** Electrical and electronic engineering, Energy storage

## Abstract

Introduction of superconductor to magnetic levitation (maglev) trains greatly enhances the performances compared to those of normal conductor maglevs, e.g. from 430 km/h of the Transrapid (in Shanghai) to 603 km/h of the L0 Series in Japan. However, one of the important constraints on development of superconducting maglevs is limited wireless feeding power for on-board superconducting magnets and cryogenic cooling. In this paper, a persistent-current superconducting magnets system with solid nitrogen (SN_2_) cooling preservation is proposed for liberation of its demanding on-board power feeding requirement. The magnets are optimally designed with no-insulation technique guaranteeing a safe operation with magnetic field over 0.8 T. Lasting time of persistent current (at 96.5% magnetic field retained) and SN_2_ cooling preservation (up to 40 K) is all >9 h, covering a maglev traveling distance of >5400 km at average designed speed of >600 km/h. The magnets have anti-vibration ability of 15 g (147 m/s^2^) up to 350 Hz, which has covered the vibratory motion range in maglevs. This work is intended to provide a reference for superconducting maglev developments.

## Introduction

High-speed railways such as CRH, TGV, ICE, Shinkansen, and KTX, are running worldwide providing people with convenience. However, it is difficult to operate trains when speed is over 500 km/h due to the rail-wheel propulsion and catenary/pantograph power feeding system^1^. To acquire faster speed, relative research programs on magnetic levitation (maglev) train have started since the first publication^2^. A world record of ultrahigh speed at 603 km/h was made by the L0 Series superconducting maglev in Japan in 2015^3^. The introduction of the superconducting technology to the maglevs is straightforward, to substantially enhance its performance because a superconducting magnet can easily provide magnetic field well above that possibility with a conventional permanent magnet, i.e., >0.5 T at centimeters even a decimeter away from magnet surface, while keeping a compact volume and light weight.

High-temperature superconducting (HTS) materials show great advantages on higher critical current density (*J*_c_), critical magnetic field (*B*_c_) and other performances in comparison to low-temperature superconducting (LTS) materials (e.g. Nb-Ti superconductor)^4–9^. Different from the LTS materials that are operated in 4.2 K liquid helium (LHe) bath, HTS materials have much higher critical temperature (*T*_c_), which provide possibility of LHe-free and safe operation in cheaper liquid nitrogen (LN_2_) bath at 77.2 K with large thermal margin. Besides, the second generation (2G) HTS wires (e.g. YB_2_C_3_O_7-δ_) have advantages over 1G wires (e.g. Bi_2_Sr_2_Ca_2_Cu_3_O_x_) including higher in-field *J*_c_ and enhanced mechanical property. Commercial 2G wires allow minimum bending diameter of 10 mm and maximum axial tensile stress of 427 MPa with >95% *J*_c_ retention^10^, which are satisfying in maglev applications. It is possible to optimally design HTS magnets for certain usages, and energize them conveniently by external current sources, persistent-current switches (PCSs), or flux pumps with high efficiency^11–14^. It is validated that on-board 2G HTS magnets are able to operate well for maglevs at rated speed of 620 km/h^15^. 2G HTS materials are promising in the superconducting maglev application.

However, obstacles are remained. Maglevs remove all the physical contacts to the ground to maximally reduce running frictions, thus, the kW-scale wireless power feeding is quite limited when considering the energy consumption during the operation of the on-board superconducting magnets including cooling and energizing with cryocoolers, compressors, large-current sources and sometimes water chillers besides the power for carriages. Attempts have been made by us for realization of persistent-current superconducting magnets without power source^16^. And solid cryogen auxiliary cooling for possible cryocooler-free operation is also investigated by groups^17,18^.

Inspired by the aforementioned advantages and limitations, the first demonstration of an on-board persistent-current superconducting magnets system with cooling-power-free operation especially for superconducting maglevs, is proposed in this work. Therefore, cooling and energizing facilities can be removed from train carriages. For the magnets system, designs combining electrical, mechanical, and thermal aspects are reported mainly including optimization of no-insulation magnets, analysis of field and energizing characters, performance of persistent-current mode and strategies for enchantment of anti-vibration and cooling abilities. This paper is aimed at providing detailed designs and analysis of the on-board superconducting magnets system, and the methods can be transplanted to superconducting generators for wind turbines or NMR/MRI, etc.

## Theory/Method

### T-A formulation

**Figure 1:**
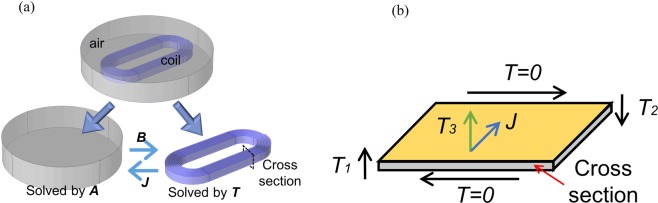
Solving process by the *T–A* formulation. (**a**) A 3D model includes air and sheet-like HTS domains. The domains are exchanged by current density ***J*** and magnetic flux density ***B*** with the two variables being solved separately. (**b**) A single turn of an HTS wire with current vector potential ***T*** actually normal to its surface. Two boundaries of the cross section are set by *T*_1_ and *T*_2_ (i.e., the components of ***T***).

To date, 3D modelling of 2G HTS wires is still challenging due to intensive computation density caused by high aspect ratio (usually in 10^3^ scale) of the wire even with artificial scaling-up^19^. An efficient 3D model based on the *T–A* formulation is proposed, tackling the high aspect ratio problem by a sheet approximation^20^. Superconducting and non-superconducting domains are solved with *T* (current vector potential) and *A* (magnetic vector potential) formulations, respectively. The process of *T-A* formulation is depicted in Fig. 1a.

The formulation is deduced on the framework of Faraday’s law being applied to the superconducting sheet:

1 $$\nabla \times {\boldsymbol{E}}=\nabla \times ({E}_{0}{(\frac{|{\boldsymbol{J}}|}{{J}_{c}(B,\theta )})}^{n}\frac{{\boldsymbol{J}}}{{J}_{c}(B,\theta )})=\nabla \times ({E}_{0}{(\frac{|\nabla \times {\boldsymbol{T}}|}{{J}_{c}(B,\theta )})}^{n}\frac{\nabla \times {\boldsymbol{T}}}{{J}_{c}(B,\theta )})=-\,\frac{\partial {\boldsymbol{B}}}{\partial t}$$

where ***B*** and ***E*** denote the magnetic flux density vector and electric field vector. For 2G HTS materials, ***E*** and current density vector ***J*** are depicted by the *E–J* power law. Index number *n* describes resistance incensement of superconducting material, which is 27 from supplier Shanghai Superconductor Technology Co., Ltd (SSTC). *E*_0_ is 10^−4^ V/m criterion of quench, and *J*_*c*_*(B, θ)* is the field and angular-dependent critical current density^21^. Here in the sheet, ***J*** is constrained to flow within the superconductor, thus, current vector potential ***T*** is actually normal to the wire’s surface, as show in Fig. 1b. Next step is to replace ***B*** in Equation 1 by magnetic vector potential ***A*** based on the Ampere circuital theorem. The *A* formulation is selected for its high efficiency in solving the magnetic field^22^.

2 $$\nabla \times {\boldsymbol{B}}\mathop{=}\limits^{{\boldsymbol{B}}=\nabla \times {\boldsymbol{A}}}\nabla \times \nabla \times {\boldsymbol{A}}={\mu }_{0}{\boldsymbol{J}}$$

By now, the *T-A* formulation is established. Current *I* to the magnet is an integral of the current density ***J*** over the cross-section *S* of the wire. Since the ***T*** is normal to the wire surface, thus:

3 $$I={\iint }_{S}{\boldsymbol{J}}dS={\iint }_{S}\nabla \times {\boldsymbol{T}}dS=({T}_{1}-{T}_{2})\cdot d$$

where *T*_1_ and *T*_2_ are components of ***T*** on the left and right edges, respectively (see in Fig. 1b). And *d* is 1 μm (i.e., real thickness of the wire). Furthermore, combining with homogenization technique which models a stack of HTS wires as a homogeneous anisotropic bulk^21^, *T–A* formulation can lead to an even faster calculation with agreeable modelling results.

### Design optimization of no-insulation (NI) magnets

**Figure 2:**
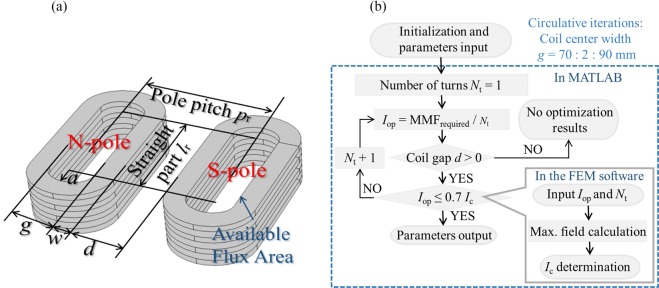
Design optimization of the HTS magnets, including (**a**) design constraints and (**b**) optimization process. The optimization process preferentially control *N*_t_ since the increasement of it means more HTS wires consumed. The optimization occurs when *I*_op_ = 0.7*I*_c_. The design optimization is implemented through MATLAB combining with COMSOL.

In the proposed system, design optimization of a pair of magnets is conducted. The design is to seek an optimal dimension and size of the magnets generating needed direct current (DC) magneto-motive force (MMF) with the minimum HTS wires length on the premise of safe operation. Several same double pancake (DP) superconducting coils with no-insulation (NI) technique, which remove the turn-turn insulations, are adopted in the magnets system for the following benefits: compactness for reduced magnet volume installed under the train carriages and stability for quench current bypassing through turns without devastating heat^23–25^. The optimization can be expressed mathematically with an objective function of the minimized HTS wires length *L*_wire_ and corresponding geometrical and physical constrains:

minimize:

4 $${L}_{{\rm{wire}}}=f({\rm{{\rm X}}})$$

subject to

5 $$\{\begin{array}{c}MMF={I}_{{\rm{op}}}\cdot {N}_{{\rm{t}}}\ge MM{F}_{{\rm{target}}}\\ {I}_{{\rm{op}}}\le 0.7\cdot {I}_{{\rm{c}}},\,\,{I}_{{\rm{c}}}={g}_{2}({\rm{{\rm X}}},B)\\ 2\cdot a=\alpha g,\,{\rm{{\rm X}}}=[{l}_{{\rm{r}}},g,a,w,d,{p}_{{\rm{r}}}]\\ w={T}_{{\rm{HTS}}}\cdot {N}_{{\rm{t}}}/(2\,n)\\ {p}_{{\rm{r}}}=260,{l}_{{\rm{r}}}=200\\ w > 0,g > 0,d > 0\end{array}$$

where *I*_c_ is critical current, which is influenced by coil size X and magnetic field *B*, and *B* includes self and external magnetic field. Operating current *I*_op_ is limited by *I*_c_, or critical current density *J*_c_. Here *I*_op_ of the magnets is set ~0.7*I*_c_ for a safe margin^1^. *N*_t_ is sum of turn numbers of the magnets, *T*_HTS_ is wire thickness, and *n* is total number of DP coils consisted in the magnets. Geometrical parameters including straight part *l*_r_, coil center width *g*, minor axis *a*, winding width *w*, coil distance *d*, and pole pitch *p*_r_, are shown in Fig. 2a, where *l*_r_ and *p*_r_ are predetermined for required available flux area. For semielliptical curving parts, 2*a* is usually set at least *α* times of *g* for avoidance of an over-bent damage to 2G HTS wires in the junction of straight and curving part (usually *α* is 0.75, or 1 for semicircular curving part).

Besides the required MMF which only relates to *I*_op_ and *N*_t_, magnetic field quality should also be taken into consideration. Among the geometrical parameters, *g* (or *d*) is independent, providing chances to alternate magnetic field quality under given MMF. Take *g* for analysis, too large or too small of its value both leads unsatisfying results, that is, too much wires wasted in the curving parts or too degraded *I*_c_ due to concentrated field at the corners. Especially for the condition that *g* is too small, *I*_op_ is limited to a low value which means large *N*_t_ is required for the needed MMF. As a result, objective function of the optimization is not met. Moreover, a too-small *g* causes magnetic field distorting from sinusoidal wave. Therefore, an optimization process considering the balance of the MMF and field quality, is shown in Fig. 2b. In the process, safe margin of 0.7 rather than the required MMF is set to be a judgment condition, thus, *I*_op_ can be calculated by a certain *N*_t_ under the given target MMF. Circulative iteration of *I*_op_ is therefore avoided. For quick acquirement of optimization results, circulative iterations of parameters are intended to be minimized only for *g*. And *I*_c_ of the magnets is analyzed by a three dimensional finite element model in a simulation software COMSOL. For the *I*_c_ determination in field of engineering application level with large size coils (i.e., in hundreds of turns), load-line method is also widely adopted as well as the *T-A* formulation for both convenient analysis and acceptable precision^26,27^.

### NI magnets with individual PCS

**Figure 3:**
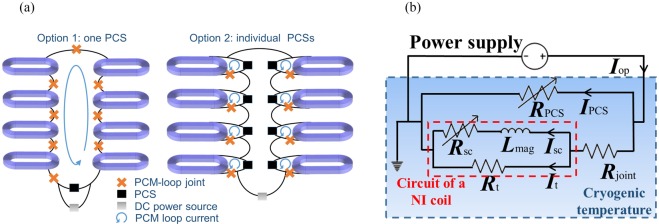
(**a**) Operations of PCS, i.e., one PCS for the whole or individual PCSs for each DP coil. And (**b**) the equivalent circuit model for a NI DP coil with its PCS. In the cryogenic environment, PCS is depicted by a temperature-dependent variable resistance *R*_PCS_. The lower branch has two parts, the left one is circuit of a NI coil^25^, and the right one is a joint (resistance *R*_joint_) of two coil terminals. *R*_t_ is the turn-turn resistance mainly controlled by winding tension and surface treatment of HTS wires. *R*_SC_ is superconducting resistance depicted by *ρ* = *E*/*J*, where *E* and *J* relate as *E–J* power law (in Equation 1). *L*_mag_ is the magnet inductance.

During on-board serving, the magnets form a closed superconducting loop by connecting the terminals as a joint. Current can maintain in the loop in persistent-current mode (PCM). However, flux creeps and joint resistance slowly dissipate the in-loop current. Fortunately, joint resistance has been reduced to a <10 nΩ scale and even to superconducting in some conditions^28^. A closed superconducting loop makes the magnets impossible to be energized using only a power supply connected across such a shorted loop without a PCS. The PCS “opens” the closed loop by a transition from its superconducting state to normal state (i.e., resistive state) when any one of three factors: temperature *T*, current density *J*, or magnetic field *B*, exceeds a certain critical value^29^. Wherein the heat-triggered PCS controlled by temperature is adopted here for its operation and facility friendly features. In PCM, the DP coils in the magnets can form a large whole loop by 1) one PCS or respective loops by 2) individual PCSs, as shown in Fig. 3a. One PCS is simple but risky because accidental quench of a DP coil may seriously influence the other ones and finally lead to current dissipation in all coils. As for individual PCSs, each independent current circulation shares operating risk^30^. And switches of the PCSs are unnecessary to be strictly simultaneous. Given compromise between reliability and complexity, option 2 is used.

An equivalent circuit model is established for a NI DP coil with its PCS, as shown in Fig. 3b. The aforementioned enhanced stability of a NI magnet is more visualized: current bypasses the local quench (where the *R*_SC_ is large) though the *R*_t_ branch, a natural protective by-path, and usually *R*_t_ is in ∼50 µΩ·cm^2^ scale with copper stabilizing layer at pressure of 5 MPa^31^. The governing differential equations of the model are

6 $$\{\begin{array}{c}{I}_{{\rm{PCS}}}{R}_{{\rm{PCS}}}=({I}_{{\rm{op}}}-{I}_{{\rm{PCS}}}){R}_{{\rm{joint}}}+({I}_{{\rm{op}}}-{I}_{{\rm{PCS}}}-{I}_{{\rm{SC}}}){R}_{{\rm{t}}}\\ {L}_{{\rm{mag}}}(d{I}_{{\rm{SC}}}/dt)+{I}_{{\rm{SC}}}{R}_{{\rm{SC}}}=({I}_{{\rm{op}}}-{I}_{{\rm{PCS}}}-{I}_{{\rm{SC}}}){R}_{{\rm{t}}}\\ {R}_{{\rm{PCS}}}={\rho }_{{\rm{stab}}}(T)\cdot {l}_{{\rm{PCS}}}/{A}_{{\rm{stab}}}\end{array}$$

where *I*_PCS_ and *I*sc denote the current in PCS and in magnets. *l*_PCS_ is length of PCS. Resistance of superconducting layer increases much faster than that of copper when temperature of PCS is heated above the *T*_c_, therefore, *ρ*_stab_(*T*) and *A*_stab_ are temperature-dependent resistivity and total cross section area of copper laminated layers of an HTS wire, respectively. Other parameters are explained in the description of Fig. 3b.

### Solid nitrogen cooling preservation

Cooling power has to be preserved in advance to realize the power-free operation in traveling. High *T*_c_ of the 2G HTS materials, specifically, like the YB_2_C_3_O_7-δ_ wires adopted in this system, provides a possibility of using inexpensive, lightweight, electrically insulating, and nontoxic solid nitrogen (SN_2_) as an excellent thermal mass enhancer or a “thermal battery”. SN_2_ is an attractive coolant when combined with a cryocooler, because 1) SN_2_ has a jump of the value of thermal conductivity at phase transition (from β-SN_2_ to α-SN_2_) temperature of 35.6 K^32,33^, 2) latent heat of 8.3 J/cm^3^ during SN_2_ phase transition provides extra cooling energy^17^, and 3) excellent heat capacity of SN_2_ (enthalpy density of 15 kJ/m^3^ @ 4.2–4.5 K) is ∼50 times that of copper. To take advantage of thermal conductivity enhancement and latent heat in the α-β phase transition, operating temperature of the magnets system is determined at ~30 K, which also provides sufficient cooling margin for SN_2_ remaining solid up to 63.2 K.

**Figure 4:**
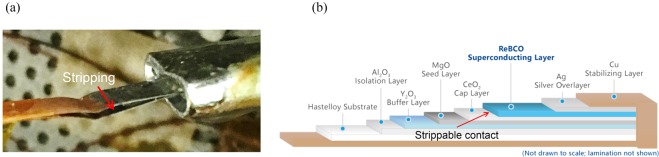
(**a**) Stripping happens to the YB_2_C_3_O_7-δ_ wire terminal immersed in SN_2_ in Ling’s experiemnt^30^ due to different thermal contractions. (**b**) A typical structure of an HTS wire^40^. The stripping usually happens between ReBCO (Re = rare earth) layer and CeO_2_ layer, which are mechanically vulnerable.

However, SN_2_ has relatively large thermal contraction coefficient (i.e., 3% from triple point of 63.2 K to 10 K) compared to those of copper and stainless steel (i.e., 0.02–0.03% from 65 K to 10 K), which is risky for mechanically vulnerable parts like terminals and joints of single-turn HTS wires immersed in SN_2_ without protection, as shown in Fig. 4. Therefore, HTS wires with copper and tin package are selected for avoidance of the unexpected damage. Under transverse tensile stress, the wires have delamination strength of 45 MPa at room temperature and over 130 MPa at cryogenic temperature (from the SSTC). Another issue is poor thermal contact between HTS wires and SN_2_ if porous structure of SN_2_ crystal is generated by decompression or too-fast cooling^33,34^. Meanwhile, SN_2_ is possible to separate from wire surface (i.e., the dry-out phenomenon) when thermal disturbances pass over, for example, 18 times^34^, and lead to increment of surface thermal resistance. Countermeasure of cooling plates setting between adjacent DP coils and bobbins, respectively, is proposed in this work to improve thermal contact. This “sandwich” array of cooling plates made of high thermal conductivity material guarantee multidirectional cooling pathways from SN_2_ to the immersed magnets, namely, through direct connection from SN_2_ and conductive delivery from cooling plates.

## Results

The proposed HTS magnets system has three key points: (1) magnet performances for the maglevs, (2) persistent-current mode, and (3) SN_2_ cooling preservation. Results are conducted from the above points in electrical, mechanical and thermal aspects.

### Manufacture and magnetic & electrical performances

**Figure 5:**
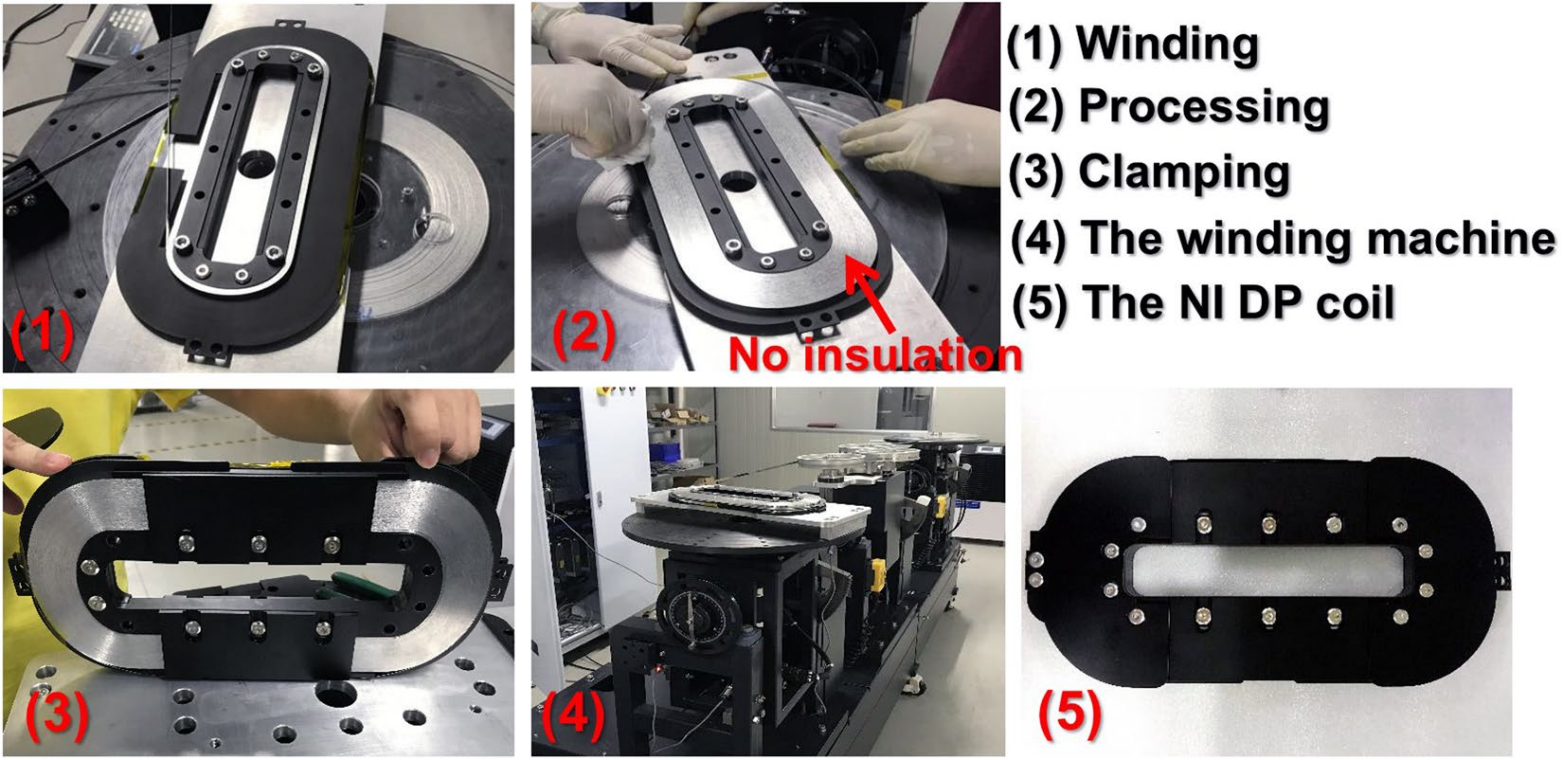
Manufacture of a 300-turn NI DP coil. The bobbin is made of high thermal conductivity aluminum alloy 6063-T5 with hard anodizing surface treatment for formation of thin (~70 μm) but reliable insulations.

Design optimization results are listed in Table 1, where MMF is predetermined to 360 kA @ 30 K according to our design specifications of a maglev^35^. Manufacture of a NI DP coil is shown in Fig. 5. Note that winding tension of 50 N is maintained by four clampers, because the winding tension is closely related to self-protection ability and electrical performances of the magnets with NI technique^36^. And epoxy resin impregnation is not adopted here because SN_2_ has thermal conductivity nearly at least an order of magnitude better than that of epoxy at cryogenic temperature^17^.

**Table 1:** Summary of the design optimization results at 30 K and 77 K.

Parameters	Specifications
Geometrical size *g/w/d/l*_r_*/a/p*_r_ (mm)	86/36/102/200/32.3/260
Operating current @ 30 K/@ 77 K	300 A/48 A
Magnet *I*_c_ @ 30 K/@ 77 K	387.46 A/68.14 A (self-field)
MMF per magnet @ 30 K/@ 77 K	360 kA/57.6 kA
HTS wire length per magnet	~910 m
Mass per magnet (including bobbins)	~18 kg
Number of NI DP coils per magnet	4
Total inductance of the two magnets	551.11 mH

**Figure 6:**
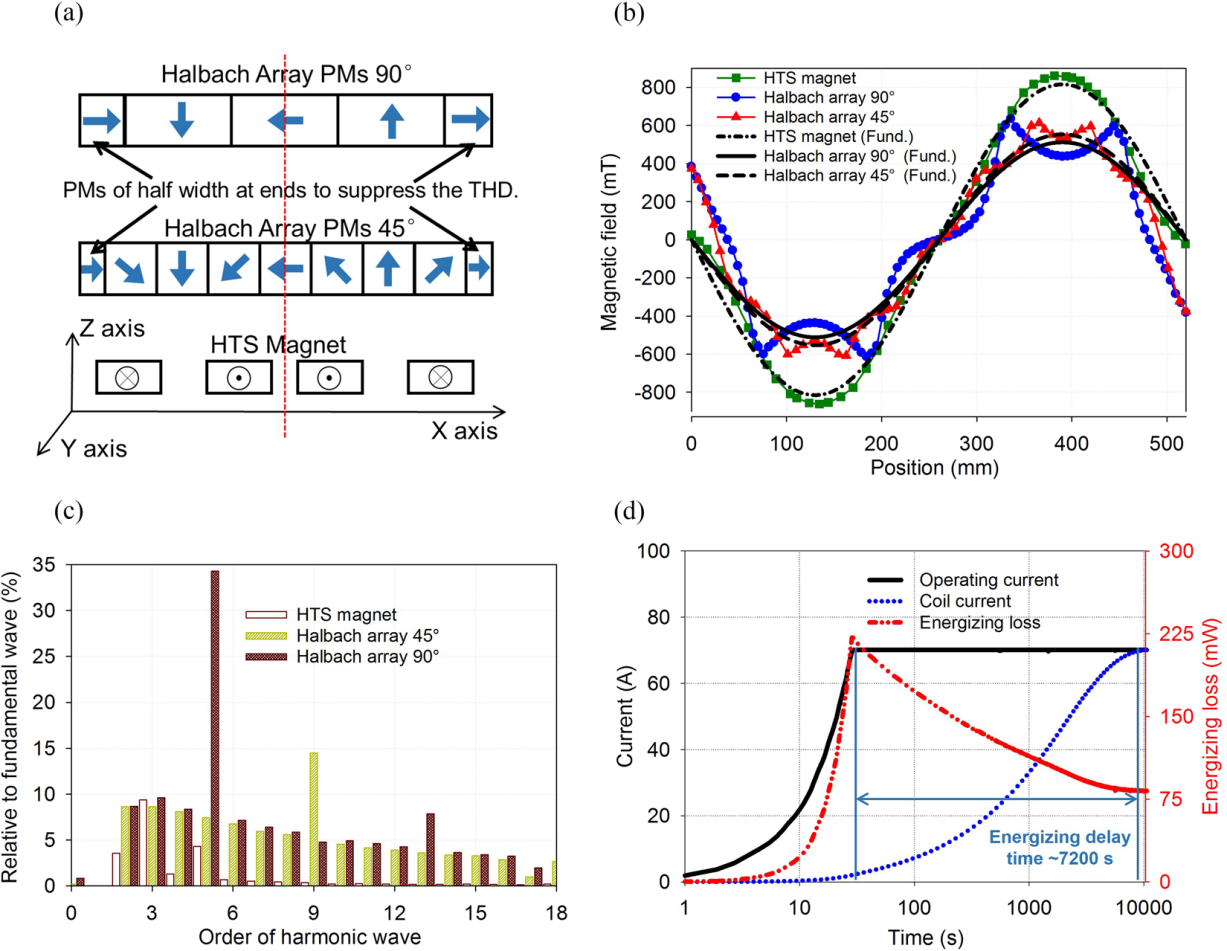
Magnetic and electric performances of the designed magnets. (**a**) The HTS magnets and the Halbach array PMs with magnetization angle of 90° and 45°. Height (Z axis) and length (Y axis) of the Halbach arrays are consistent with the HTS magnets height (including bobbins) and straight part length *l*_r_. (**b**) Magnetic field waveforms and their corresponding fundamental components of the HTS magnets and PMs. (**c**) Harmonic analysis of the magnetic fields. (**d**) Energizing characters of the HTS magnets including energizing delay and loss.

MMF and magnetic field quality of the magnets are both important for propulsive force of the maglevs, because the former dominates the force magnitude and the latter closely influence output smoothness of the force. The HTS magnets are compared to commercially available permanent magnets (PMs) with the strongest field, i.e., N52 NbFeB PMs with remnant field of 1.45 T. Then PMs are set in Halbach array for further enhancement of magnetic field. Only in this way advantages of the HTS magnets over the PMs can be proved. Both the HTS magnets and the PMs are set one pair of poles as shown in Fig. 6a. Magnetic field waveforms and their corresponding fundamental components at 30 K temperature at 50 mm gap above the magnets are analyzed by fast-Fourier-transformation (FFT), as shown in Fig. 6b. The field generated by the HTS magnets (~803 mT at peak) is ~1.45–1.5 times that of the PMs. Harmonic analysis of the fields is shown in Fig. 6c. Total harmonic distortions (THD) of the fields generated by the HTS magnets, the PMs with an angle of 45° and 90° are 5.98%, 28.41%, and 43.04%, respectively. Results indicate that field of the HTS magnets have advantages both in amplitude and quality. As for the electrical performances, extra consideration of energizing characters regarding to the NI magnets should be pointed out, which include energizing delay and additional energizing loss, relating to practicability of commercial running of the maglevs and evaluation on cooling capacity of SN_2_. The additional loss and delay are caused by current leakage occurring on turn-turn contact resistance (i.e., the *R*_t_ branch in Fig. 3b) due to magnet inductance. Test results of above energizing characters of a NI DP coil is shown in Fig. 6d. The whole energizing process starts with current source ramping rate of 2.33 A/s, and finishes in ~2 h with 99% MMF achieved. Because of *RL* characters of NI technology as described in Equation 6, MMF can never be energized to target value. Maximum energizing loss attained in the energizing process is ~225 mW, and average value is ~96.7 mW. *R*_t_ is measured to be 21.94 *μ*Ω, or calculated from measured value to be 3.32 *μ*Ω·cm^2^ ^31^. Table 2 lists energizing characters of the magnets under different current source ramping rate. The energizing time is controlled to ~3.1 h, which is acceptable in engineering.

**Table 2:** Energizing characters of the HTS magnets.

Ramping rate (A/s) to 300A	0.5	1	2	5	10
Energizing time (s)/(h)	11214/3.12	11060/3.07	10984/3.05	10938/3.04	10924/3.03
Max. leak current (A)	269.50	284.15	291.88	296.65	298.27
Avg. energizing loss (W)	2.06	2.16	2.217	2.25	2.26
Max. energizing loss (W)	14.53	16.15	17.04	17.60	17.79

### Persistent-current switch

**Figure 7:**
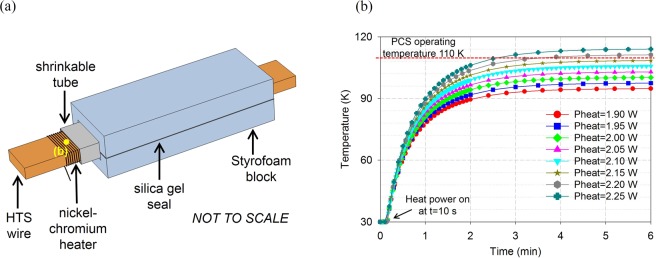
The heat-triggered PCS is converted from a 70 mm section of the HTS wire. (**a**) Structural drawing of the PCS. Note that the nickel-chromium heater and shrinkable tube are actually enclosed in the Styrofoam heat “trap”. The temperature point on the PCS shows that (**b**) the PCS is opened within 5 min by 2.20 W heat power.

Individual PCSs (or modularization^17^) are chosen. The PCS design have to take electrical and thermal performances into consideration. The PCS is converted from a section of an HTS wire, as shown in Fig. 7a. Normal-state resistance of the PCS should be large enough with its bypass current (i.e., *I*_PCS_ in Fig. 3b) <10% of the coil current^37^. For this magnets system, *I*_PCS_ is limited to <5 A, thus the minimum required *R*_PCS_ can be calculated by the voltage *V* across the magnets at an average ramping rate of 0.027 A/s (i.e., 300 A/3.1 h).

7 $${R}_{PCS}\ge \frac{V}{5\,\,{\rm{A}}}=L\frac{dI}{dt}/5\,{\rm{A}}\approx 3\,{\rm{m}}{\rm{\Omega }}$$

When the PCS is heated to ~110 K, the copper laminated layers mainly conduct current because superconducting layer has already quenched to large resistance. With copper resistivity of ~5.2 nΩ·m at 110 K^37^ and cross-sectional area of 9 × 10^−7^ m^2^, the total section length for 8 PCSs is ~560 mm (or ~70 mm for each one).

Heat of each PCS is “trapped” in two Styrofoam (a trademarked brand of closed-cell extruded polystyrene foam) blocks pressing on both sides of the PCS, as shown in Fig. 7a. Styrofoam has quite low thermal conductivity, and has slight elasticity without cracks at cryogenic temperature. In the Styrofoam heat trap, a close-wound nickel-chromium heater is frapped by a shrinkable tube for a good thermal contact to the wire. Then the PCSs are located above the SN_2_ level rather than immersed in it to further limit the heat load to cryogenic environment. Different from evaporative cooling of LHe, SN_2_ vapor pressure is <10^−10^ Pa @ 10–15 K and <10^−5^ Pa @ 25–35 K, which means convective heat load is negligible. For each PCS, conductive heats are estimated in two pathways, that is, to SN_2_ along the wire volume (*Q*_*C*1_) and to Styrofoam on the wire surface (*Q*_*C*2_):

8 $$\{\begin{array}{rcl}{Q}_{C1} & = & 2{\lambda }_{{\rm{copper}}}\frac{{T}_{{\rm{H}}}-{T}_{{\rm{L}}}}{{l}_{{\rm{copper}}}}{A}_{{\rm{wire}}}\approx 1.92{\rm{W}}\\ {Q}_{C2} & = & 2{\lambda }_{{\rm{foam}}}\frac{{T}_{{\rm{H}}}-{T}_{{\rm{L}}}}{{d}_{{\rm{foam}}}}{A}_{{\rm{copper}}}\approx 0.13{\rm{W}}\end{array}$$

where *λ* is average thermal conductivity of copper and Styrofoam at 30 (*T*_L_) −110 (*T*_H_) K (~800 W/mK and 0.01 W/mK, respectively^37^). Area *A*_wire_ is 0.25 mm wire thickness × 6 mm wire width, and *A*_copper_ is 70 mm PCS length × 6 mm wire width. *l*_copper_ is wire length from one PCS terminal to the SN_2_ level (the PCS is not immersed in SN_2_), which is ~100 mm. *d*_foam_ is thickness of one Styrofoam block, and *d*_foam_ = 5 mm is sufficient here. Maximum radiative heat load is:

9 $${Q}_{R}=2{A}_{{\rm{copper}}}\varepsilon \sigma ({{T}_{H}}^{4}-{{T}_{L}}^{4})\approx 0.03\,{\rm{mW}}$$

where ε is emissivity of copper at 110 K (~0.004^38^), and σ is Stefan-Boltzmann constant. Therefore, total heat load of each PCS is ~2.05 W, however, its actual value in practice may be larger because of imperfect thermal insulation. Figure 7b shows that 2.15–2.20 W of actual heat power fully opens the PCS to 110 K within 5 minutes.

### Vibration and PCM performance

**Figure 8:**
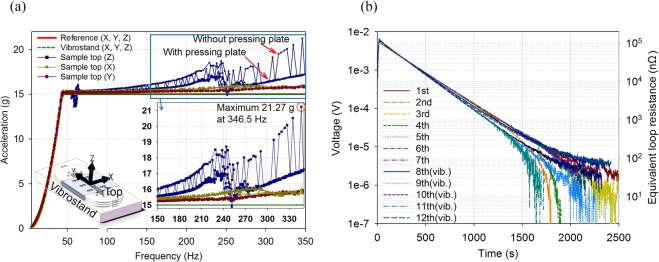
Mechanical and electrical properties in the vibration test. (**a**) The acceleration in Z-direction increases when frequency is >250 Hz. A pressing plate (i.e., the cooling plate in the proposed system) is recommended to suppress the acceleration increasement. (**b**) Loop resistance stabilizes at <75 nΩ no matter whether it is vibrated or not, and corresponding persistent-current lasting time is >9.08 h.

Different from static magnets like those in NMR/MRI, the on-board magnets deliver propulsive force to carriages from the ground coils, and sometimes with stray forces due to harmonic waves. The forces are able to cause strong vibrations and may influence performances of the magnets mechanically and electrically. Therefore, according to the running speed, the pole pitch, and the precedent^39^, vibration tests for over 6 hours with maximum of 350 Hz in frequency and 15 g (~147 m/s^2^) in acceleration are directly delivered to the coil to simulate mechanical environment in ultrahigh speed running. Figure 8a shows test results of actual acceleration changes in X, Y, and Z directions. Amplification of acceleration over 21 g is found in Z direction when frequency is close to 350 Hz near resonance point. A pressing plate covering the coil surface is suggested to suppress the unexpected acceleration amplification. Here the aforementioned aluminum alloy “sandwich” cooling plates are the double benefits for multidirectional cooling and acceleration suppression. Figure 8b shows electrical property regarding to PCM, which is the most essential item in the vibrations. Resistance of the superconducting loop is an effective reflection of PCM performance since the resistance relates directly to the in-loop current decay. The equivalent loop resistance firstly rises due to current leakage in turn-turn by-path, then gradually drops (i.e., energizing delay). The loop resistance stabilizes at <75 nΩ (mainly due to joints) in PCM no matter whether it is vibrated or not. Therefore, the anti-vibration ability of this design of HTS coil is verified. Lasting time *t*_L_ of 96.5% persistent current retained can be estimated by *R-L* decay curve and time constant *τ* described below, where the 96.5% is derived from our acceptance outline.

10 $$\{\begin{array}{rcl}{e}^{-{t}_{{\rm{L}}}/\tau } & = & 96.5 \% \\ \tau  & = & L/{R}_{{\rm{loop}}}\end{array}$$

where *L* is inductance of the magnets (0.551 H) and *R*_loop_ is total loop resistance (<600 nΩ). *t*_L_ is calculated to >9.08 h. With average designed speed of >600 km/h, total traveling distance of a maglev is >5400 km without stopping.

### Cryostat and SN_2_ cooling preservation

**Figure 9:**
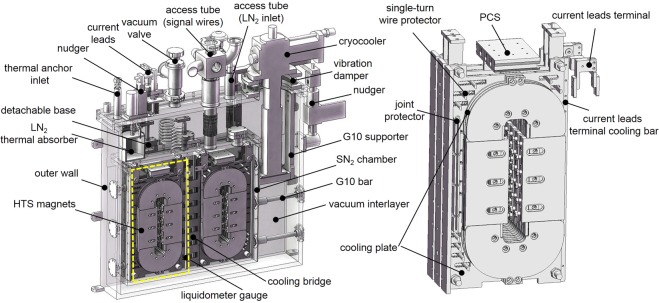
The magnets system. The HTS magnets are installed in a SN_2_ chamber. Vacuum interlayer significantly reduces convective heat load from room temperature. Radiative heat load is reduced to 1/16 of its original level by 15 layers of radiation screens wrapping outside the SN_2_ chamber. Conductive heat load is minimized by hollow epoxy resin G10 bars with low thermal conductivity of 0.2 W/mK @ 30 K and 0.7 W/mK @ 300 K^37^. Access tubes made by bellows are used to suppress conductive heat load by increasing length of heat pathway. The SN_2_ chamber and its flange are all made of aluminum alloy (Al 6063-T5) for three reasons: (1) avoidance of unexpected leakage at cryogenic temperature due to different thermal expansion coefficients of dissimilar materials^18^, (2) ~1/3 mass density that of copper, and (3) good thermal conductivity (~250 W/mK @ 30 K) for direct connecting to a cryocooler. Besides, the current leads and the cryocooler are detached automatically by two nudgers when energizing process finishes.

For an easier observation of interior structure, a 60% cross-sectional figure of the magnets system is shown in Fig. 9 with all the details. During the design, it is found that heat load in current leads depicted by the equation below, accounts for a high proportion (~45.5%) of total heat loads even though with optimal size.

11 $${P}_{{\rm{current}}{\rm{leads}}}=2({I}_{{\rm{Cu}}}^{2}{\rho }_{{\rm{Cu}}}l/A+{\lambda }_{{\rm{Cu}}}A({T}_{{\rm{RT}}}-{T}_{{\rm{L}}})/l)$$

where *I*_Cu_ is the rated current in the leads (i.e., 300 A), *ρ*_Cu_ and *λ*_Cu_ are average electrical resistivity and thermal conductivity of copper (RRR = 50) at 30 (*T*_L_) −300 (*T*_RT_) K (8.154 nΩm and 502.1 W/mK, respectively^37^). *l* and *A* are total length and cross-sectional area of one current lead. Diameter of 6 mm (or current density of ~10 A/mm^2^) for each current lead is determined based on the experience from our former cryostats. It is known that too-long or too-short current leads both result a high heat load due to joule heat domination or conductive heat domination. An optimal length *l* = 0.4 m with the minimum heat load of 19.97 W for each current lead is calculated. To further suppress the heat load, a LN_2_ thermal absorber (see in Fig. 9) is connected to the warm terminals of the current leads. The heat load drops to a minimum value of 3.98 W for each when *l* = 0.6 m (*ρ*_Cu_ and *λ*_Cu_ are 1.065 nΩm and 877.1 W/mK, respectively, at 30–77 K^37^). Further strategies on reduction of heat loads including detachable HTS-copper hybrid current leads and demountable cryocooler, are also adopted. Detailed estimated heat loads and strategies are summarized in Table 3.

**Table 3:** Estimated heat loads on the HTS magnets system and corresponding optimization strategies.

Thermal load (W)	Current leads and cryocooler undetached			Current leads and cryocooler detached
	Before opt.	After opt.	Opt. strategy	
Radiative heat	5.24	2.62	Radiation screens	2.62
Residual air	0.44	0.44	N/A	0.44
Current leads	39.94	7.96	LN_2_ thermal anchor	0
Access tubes	4.50	3.40	Bellows	3.40
Epoxy resin G10 bars	10.27	4.43	Hollow structure	4.43
Cryocooler (cold head)	8.00	8.00	N/A	0
HTS magnets	2.16	2.16	N/A	0
PCS	17.20	17.20	N/A	0
TOTAL	87.75	46.21		10.89

The design optimization guarantees a 10 K margin of SN_2_ cooling preservation for persistent-current mode. More specifically, persistent current in the magnets will decay faster due to degradation of superconducting performance if ambient temperature passes over 40 K, though it does not mean any damage to the magnets. The SN_2_ chamber is filled with 11123 cm^3^ of SN_2_, which could provide enthalpy density of ~27.60 J/cm^3^ from 25 K to 40 K^37^. Besides, subassemblies like cooling plates, chamber wall, and flange have enthalpies of ~53.77 kJ at the same temperature range. Therefore, when cryocooler and current leads are detached, the total enthalpy of 360.76 kJ for a net heat input of 10.89 W can prolong a temperature rise from 25 to 40 K to a period of ∼9.2 h, which perfectly covers the PCM lasting time of 9.08 h. Even though that all the magnets are quenched at 40 K and suddenly release stored energy of 24.8 kJ, temperature rise of SN_2_ is still less than 5 K. Thus, SN_2_ still remains in solid.

**Figure 10:**
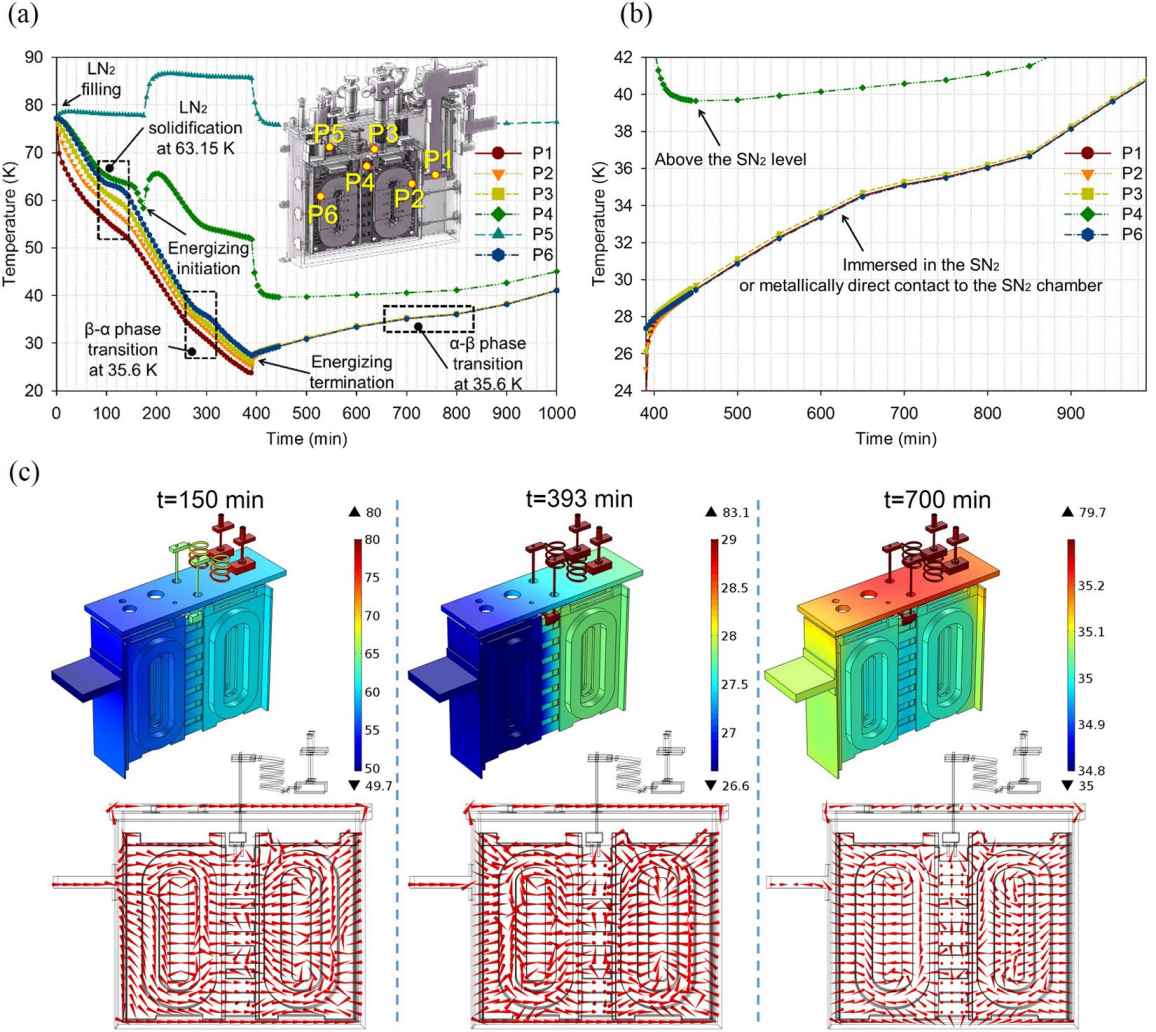
Temperature changes and cooling flux flows of the magnets system. Temperature curves at six typical locations (**a**) in the cooling-down process and (**b**) in the SN_2_ cooling preservation process. Cooling flux flows and temperature distributions at (**c**) three time points corresponding to three different conditions.

In solidification and β-α transition processes, cooling rates of 0.3 K/h and 0.001 K/s are suggested for generation of dense and crystalline SN_2_^33,34^. However, the slow cooling rates are not practical in the maglev applications. Porous and opaque SN_2_ due to a too fast cooling rate is acceptable as long as cooling preservation time and temperature uniformity on the magnets are satisfying, though the SN_2_ crystal have many defeats with lowered thermal conduction ability. Figure 10a shows the temperature changes at six typical locations in the system. The locations are pictured in the insert, that is, 1) under the cold head of the cryocooler (P1), 2) on the magnets nearest to the cold head (P2), 3) on the SN_2_ chamber flange near a access tube (P3), 4) on the current lead terminal above SN_2_ level (P4), 5) on the detachable base of the current leads (P5), and 6) on the magnets furthest to the cold head (P6). The energizing period is from t = 180 min to t = 390 min (i.e., 3.5 h), with heat load (i.e., mainly the joule heat) of the current leads increasing during this period, as temperature rise of the curves of P4 and P5. After energizing, the cryocooler is turned off to have SN_2_ cooling preservation started. Meanwhile, the cryocooler and the current leads are detached to the cryogenic environment. It takes ~9.5 h to warm the system up to 40 K, during which the maglevs are running. Two phase transitions are observed during the cooling-down process: solidification at 63.15 K (~50 min) and β-α transition at 35.6 K (~30 min), with latent heat of 25.8 J/g and 8.37 J/g, respectively^32^. However, holding temperatures of the two phase transitions are not obvious because of limited purity of the nitrogen. During the SN_2_ cooling preservation, temperature uniformity is within 1 K even for the nearest and furthest locations (P2 and P6) to the cold head (see in Fig. 10b), which prove the effect of cooling plates as thermal conductivity enhancers no matter whether the SN_2_ crystal is defective or not. Meanwhile, three different conditions of cooling flux are simulated corresponding to different temperature distributions at, for example, t = 150 min, 393 min and 700 min, respectively, as shown in Fig. 10c. Firstly, the cryocooler delivers cooling power to the magnets through the whole SN_2_ chamber. However, ~7 K of temperature difference remains. Then a quick cooling compensation (i.e., thermal equilibrium) from the coldest volumes of SN_2_ to warm volumes occurs in ~10 min after the cryocooler is off, which uniforms temperature distributions. Last, flux mainly flows outward against external heat loads because of uniform temperature in the chamber.

## Conclusions

A persistent-current superconducting magnets system with solid nitrogen cooling preservation is proposed for liberation of its demanding on-board power feeding requirement in ultrahigh speed maglevs. Firstly, the magnets are optimally designed guaranteeing a safe operation with magnetic field >0.8 T and total harmonic distortions of 5.98%, which is qualified for maglev operation. Then the magnets are wound by no-insulation 2G HTS wires for high in-field critical current, enhanced self-protective stability and volumetric compactness. Especially, persistent-current switches are carefully considered for realization of persistent-current operation of the magnets. Next, performances of the magnets system are analyzed. Electrically, lasting time of persistent-current operation of the magnets is >9.08 h (at 96.5% magnetic field retained), and corresponding traveling distance of a maglev is >5400 km without stoppings at average designed speed of >600 km/h. Mechanically, the magnets with pressing plates, which also acting as cooling plates for better thermal performance, have anti-vibration ability of 15 g (147 m/s^2^) up to 350 Hz (i.e., vibratory motion range in the maglevs) without performance degradations. Thermally, solid nitrogen provides a 9.2-hour cooling preservation period from 25 K to 40 K with satisfying temperature uniformity less than 1 K. And nitrogen remains solid even though unexpected quench of magnets happens. Conclusively, this work provides a demonstration of 2G HTS magnets system in maglev applications.

## Data Availability

The data is available for reasonable request.
